# Analyzing Knowledge Status and HIV Linkage to Care: Insights From America’s HIV Epidemic Analysis Dashboard (AHEAD) National Database

**DOI:** 10.7759/cureus.72034

**Published:** 2024-10-21

**Authors:** Oluwatoyin Ayo-Farai, Paul A Momodu, Ikenna C Okoye, Edediong Ekarika, Ifunanya T Okafor, Okelue E Okobi

**Affiliations:** 1 Jiann-Ping Hsu College of Public Health, Georgia Southern University, Statesboro, USA; 2 Medicine, International University of the Health Sciences, Basseterre, KNA; 3 Internal Medicine, Zaporizhzhya State Medical University, Zaporizhzhya, UKR; 4 Public Health, Emory University Rollins School of Public Health, Atlanta, USA; 5 Medicine, All Saints University School of Medicine, Roseau, DMA; 6 Internal Medicine, College of Medicine, University of Lagos, Lagos, NGA; 7 Family Medicine, Medficient Health Systems, Laurel, USA; 8 Family Medicine, Lakeside Medical Center, Belle Glade, USA; 9 Family Medicine, Larkin Community Hospital Palm Springs Campus, Miami, USA

**Keywords:** ahead national database, health disparities, hiv, knowledge status, linkage to care, public health

## Abstract

Background: Understanding knowledge of human immunodeficiency virus (HIV) status and timely linkage to care are crucial for improving health outcomes among individuals living with HIV. This study analyzes trends in HIV knowledge status and linkage to care using data from America’s HIV Epidemic Analysis Dashboard (AHEAD) National Database.

Methods: A retrospective database analysis was conducted utilizing the AHEAD National Database, focusing on individuals diagnosed with HIV from 2017 to 2022. Key variables included knowledge of HIV status and the percentage of individuals who received medical care within one month of diagnosis. Descriptive statistics were employed to assess trends over the years, while demographic differences were analyzed based on age, gender, race, and transmission risk.

Results: The incidence of new HIV infections in the United States has gradually declined from 37,000 in 2017 to 31,800 in 2022. Between 2017 and 2022, the percentage of people with HIV receiving medical care within one month of diagnosis increased from 77.80% to 81.60%. The actual number of individuals linked to care peaked at 27,479 in 2019 but dropped to 23,419 in 2020. However, by 2022, the number had risen to 29,753, reflecting improvements in linkage efforts. Additionally, the number of individuals aware of their positive HIV status increased from 988,546 in 2017 to 1,079,751 in 2022, with the estimated percentage reaching 87.20%. Although this indicates progress in awareness initiatives, the increase remains gradual. Disparities in care linkage across demographics, particularly among younger individuals and racial minorities, highlight the need for targeted interventions to improve overall outcomes and access to care.

Conclusions: The findings highlight significant progress in knowledge status and linkage to care among people living with HIV. Despite improvements, achieving the goal of 95% linkage to care by 2025 remains a challenge, particularly for specific demographic groups. Enhanced public health strategies and targeted outreach efforts are essential for addressing these disparities and improving overall health outcomes.

## Introduction

Human immunodeficiency virus (HIV) remains a significant global health challenge, particularly in the United States, where efforts to control the epidemic have been extensive yet continue to face barriers [1]. HIV is a retrovirus that targets the immune system, specifically CD4+ T cells, leading to their gradual depletion. This immunosuppression increases the risk of opportunistic infections and, if untreated, progresses to acquired immunodeficiency syndrome (AIDS) [1,2]. Moreover, HIV continues to pose a significant global public health challenge, having led to approximately 42.3 million deaths. As of the end of 2023, around 39.9 million individuals were living with HIV, with approximately 25.9 million (65%) located in the WHO African Region [1-4]. In 2003, approximately 630,000 people died from HIV-related causes, while 1.3 million new infections were reported [1,4]. While there is currently no cure, effective prevention, diagnosis, treatment, and care have transformed HIV into a manageable chronic condition, allowing individuals to lead healthy lives [3]. As of 2022, approximately 1.2 million people in the United States were living with HIV, with an estimated 31,800 new infections occurring annually [3,4]. The epidemiology of HIV reveals that certain populations, including men who have sex with men (MSM), racial and ethnic minorities, and those in lower socioeconomic brackets, are disproportionately affected. Understanding the distribution and determinants of HIV is crucial for developing targeted interventions that can reduce the incidence and improve outcomes [4,5].

Further, as noted above, the pathophysiology of HIV involves the virus attacking the body’s immune system, specifically CD4 cells (T cells), which are vital for immune defense. Over time, HIV can destroy so many of these cells that the body becomes unable to fight off infections and disease, leading to the development of AIDS [1,2]. Without treatment, HIV progresses to AIDS in about eight to 10 years, but with antiretroviral therapy (ART), individuals with HIV can live long, healthy lives. Early diagnosis and immediate linkage to care are critical for managing the disease, preventing transmission, and improving the quality of life for those affected [2,6,7]. Also, effective management of HIV requires that individuals not only be aware of their HIV status but also be quickly linked to appropriate medical care. Knowledge of one’s HIV-positive status is the first step in accessing treatment and care, which can suppress the virus to undetectable levels, making it untransmutable to others, a concept known as “undetectable”. Despite this, gaps in care linkage remain, particularly in populations already at higher risk of poor health outcomes [8].

The analysis of knowledge status and HIV linkage to care provides crucial insights into the effectiveness of public health interventions aimed at addressing these gaps. America’s HIV Epidemic Analysis Dashboard (AHEAD) National Database serves as a valuable resource in this regard, offering comprehensive data on HIV diagnoses, care linkage, and treatment outcomes across the United States. The database also enables the identification of trends and disparities in HIV care, informing the development of targeted interventions to improve health outcomes [9]. Therefore, this study aims to analyze data from the AHEAD database to assess the progress toward national HIV care goals. By examining the percentage of people diagnosed with HIV who received medical care within one month of diagnosis, this analysis provides a detailed understanding of the current state of HIV care in the United States and highlights areas where further efforts are needed to meet national targets

## Materials and methods

Study population and data source

The primary data source for this analysis was the AHEAD National Database, which compiles comprehensive data on HIV diagnosis, care linkage, and treatment outcomes across the United States. The AHEAD database provides annual estimates for various HIV-related metrics, including the number of people aware of their HIV-positive status and those linked to HIV medical care within one month of diagnosis [9]. Therefore, the study population included individuals newly diagnosed with HIV from 2017 to 2022. The analysis considered various demographic factors, including gender, age, race/ethnicity, and transmission category, to identify disparities in HIV care linkage and knowledge status. The data encompassed both diagnosed and undiagnosed HIV cases, allowing for a comprehensive evaluation of HIV care trends.

Variables

Two primary variables were analyzed: the percentage of individuals aware of their HIV-positive status and the percentage of those linked to HIV medical care within one month of diagnosis. The analysis also examined these variables across subgroups based on gender (male and female), age groups (13-24, 25-34, 35-44, 45-54, 55-64, and 65+), race/ethnicity (American Indian/Alaska Native, Asian, Black/African American, Hispanic/Latino, Native Hawaiian/Other Pacific Islander, and White), and transmission categories (male-to-male sexual contact, injection drug use, male-to-male sexual contact and injection drug use, and heterosexual contact).

Data collection and analysis

Data were extracted annually from the AHEAD database for 2017 to 2022. Descriptive statistics were used to summarize the percentage of individuals with knowledge of their HIV-positive status and those linked to care within one month of diagnosis. Trends over the six years were analyzed to identify changes in knowledge status and care linkage rates. Moreover, descriptive statistics that included percentages and frequencies were utilized to summarize the variables. To ascertain transparency and clarity, we ensured that the data extraction process adhered to pre-defined protocols and that consistency was maintained throughout the study years. To effectively assess the trends over six years, a longitudinal analysis was performed to assess the changes in the study population’s HIV status awareness and the correlation with the care rates over time. Additionally, subgroup analyses were conducted to assess disparities in care linkage across different demographic categories, including gender, age, ethnicity, race, and geographic location. The subgroup analysis enabled the performance of a detailed evaluation of inequities in access to HIV care.

Further, for the statistical analysis, SPSS statistical software (IBM Corp., Armonk, New York, United States) was utilized, whereby the chi-square tests were conducted to assess the correlations between the demographic factors and the outcomes of the care linkage. Also, the logistic regression models were employed to identify the predictors of effective linkage to HIV care. The study findings were further subjected to benchmarking against the 2025 national HIV care goals that target a 95% linkage to care within a month of diagnosis. Finally, we assessed the data in the context of the projected goals for a 75% decline in the new HIV infection rates by 2025 and a 90% decline by 2030 [3,10]. The comparative analysis has enabled evaluating the efficiency of existing HIV care interventions and strategies and aptly identified areas that need more targeted interventions.

Ethical considerations

This study involved de-identified, publicly available data from the AHEAD database, which did not require institutional review board (IRB) approval. The data were handled in compliance with all relevant ethical guidelines and regulations for public health data research. Although the data was de-identified, data on the patient's race was collected, mainly through self-identification by the patients. The data on the patients' races was vital to this study as it enabled the determination of HIV status knowledge and awareness rates within different racial populations and the development of different customized and targeted interventions for reaching each affected community/race. 

## Results

The incidence of new HIV infections in the United States has gradually declined from 37,000 in 2017 to 31,800 in 2022. The national goal is to reduce new HIV infections by 75% by 2025, targeting 9,300 cases, and by 90% by 2030, aiming for just 3,000 cases [3,9,10]. Despite the progress attained, significant efforts are required to meet these ambitious targets, emphasizing the importance of ongoing prevention strategies and healthcare access.

Knowledge of positive HIV status trend-based analysis.

From 2017 to 2022, the number of people with knowledge of their positive HIV status increased from 988,546 to 1,079,751. The estimated percentage of individuals aware of their HIV-positive status also showed a steady rise, moving from 848,172 (85.80%) in 2017 to 941,333 (87.20%) in 2022. This trend indicates progress in awareness efforts, although the increase remains gradual, highlighting the need for continued outreach and education to further improve knowledge and reduce stigma associated with HIV. This increase reflects ongoing efforts in HIV testing and awareness campaigns. However, the data also indicates that approximately 13% of individuals remain unaware of their HIV status, underscoring the need for enhanced testing strategies and outreach, particularly in high-risk populations. Moreover, achieving the 95% linkage to care goal by 2025 will require a focused approach that addresses the specific needs of vulnerable populations, ensures equitable access to care, and overcomes the barriers that continue to hinder timely treatment for all people living with HIV. Table 1 below presents the percentage of people with knowledge of their positive HIV status and their subsequent linkage to care. 

**Table 1:** People with knowledge of their positive HIV status -: Not available

Label	Variable	2017	2018	2019	2020	2021	2022
National data	People with knowledge of their positive HIV status	988,546	1,003,086	1,025,126	1,037,822	1,056,027	1,079,751
	Estimated percentage with knowledge of their positive HIV status	848,172 (85.80%)	861,650 (85.90%)	884,683 (86.30%)	897,716 (86.50%)	916,631 (86.80%)	941,333 (87.20%)
Based on gender	National, Male (sex)	84.80%	85%	85.30%	85.60%	86%	86.40%
	National, Female (sex)	89%	89.20%	89.50%	89.70%	89.90%	90.10%
Based on age	National, 13-24	50.50%	42.10%	44.90%	46.90%	51.10%	56.30%
	National, 25-34	70.70%	71.70%	71.50%	71.10%	71.20%	71.60%
	National, 35-44	84.80%	85.70%	85.40%	84.90%	84.60%	84.30%
	National, 45-54	92.40%	92.90%	92.70%	92.60%	92.30%	92%
	National, 55-64	-	95.60%	95.60%	95.60%	95.60%	95.60%
	National, 65+	-	97.50%	97.60%	97.70%	97.70%	97.70%

When analyzing the knowledge of HIV status based on gender, the data reveals that females consistently have a higher awareness than males. In 2022, 90.10% of females were aware of their HIV-positive status compared to 86.40% of males. This trend has persisted over the years, with females showing a stable and slightly increasing rate of knowledge. The gender gap suggests that targeted interventions may be necessary to address the specific barriers that men face in accessing HIV testing and information.

Age is a significant factor influencing the knowledge of HIV status. The data highlights a stark disparity among different age groups. In 2022, the knowledge rate among those aged 13-24 was 56.30%, a notable increase from 50.50% in 2017 but still considerably lower than other age groups. Individuals aged 25-34 showed stagnant growth, remaining around 71.60% in 2022. Older age groups, particularly those aged 55-64 and 65+, exhibited the highest rates of knowledge, with percentages reaching 95.60% and 97.70%, respectively. These findings suggest that younger individuals are less likely to be aware of their HIV status, highlighting the need for age-specific educational campaigns and testing initiatives.

**Figure 1:**
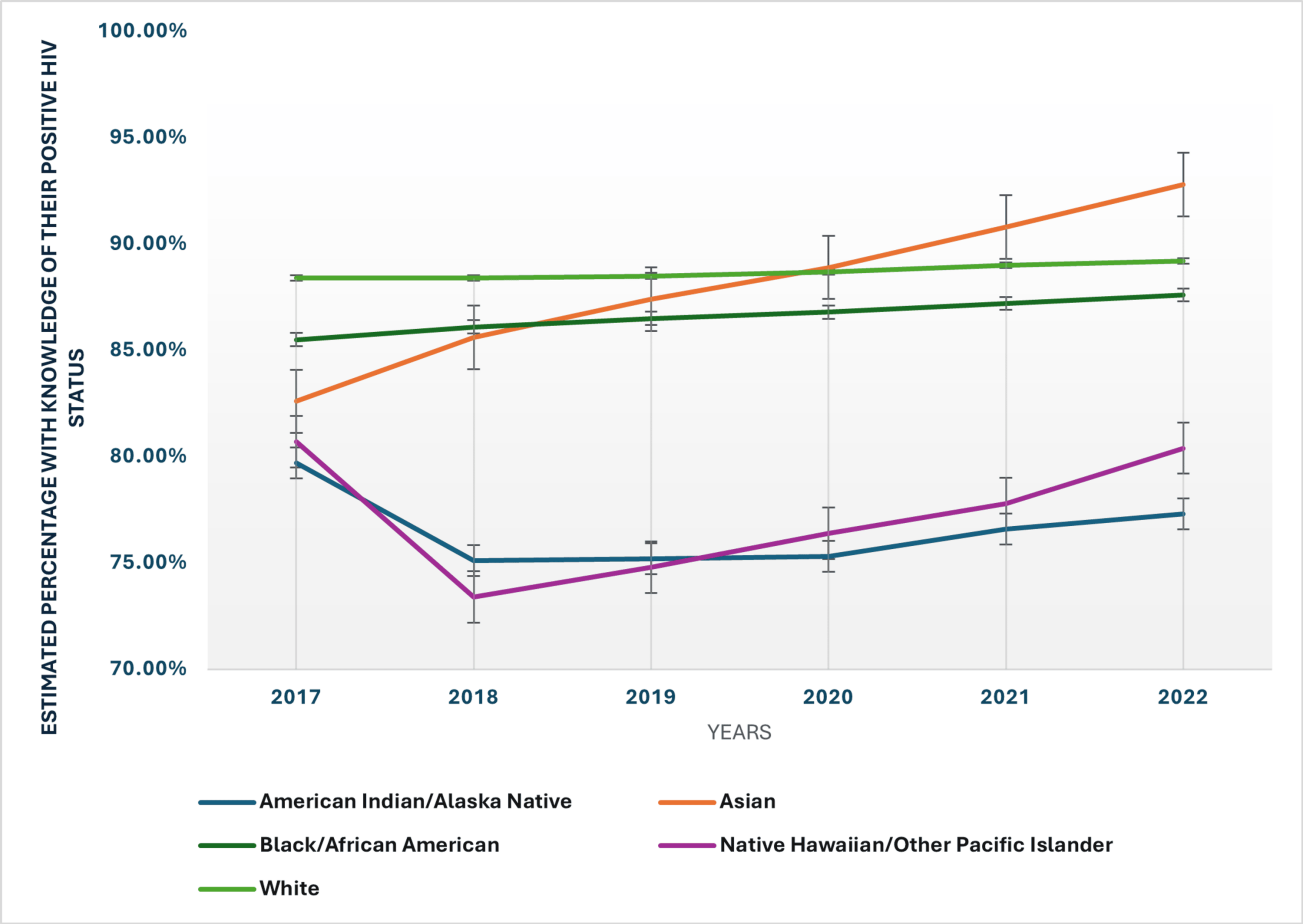
Racial and ethnic disparities in the knowledge of positive HIV status

Racial and ethnic disparities are also evident in the data. By 2022, Asians had the highest rate of knowledge of their HIV status at 92.80%, followed by Whites at 89.20%. Conversely, American Indians/Alaska Native individuals and Native Hawaiian individuals and other Pacific Islanders had the lowest rates, with 77.30% and 80.40%, respectively. The rates for Black/African American individuals and Hispanic/Latino individuals were around the national average, at 87.60% and 84%, respectively. These disparities point to the ongoing challenges in reaching certain racial and ethnic groups with effective HIV testing and awareness programs. The racial and ethnic disparities in the knowledge of positive HIV status have been presented in Figure 1.

**Figure 2:**
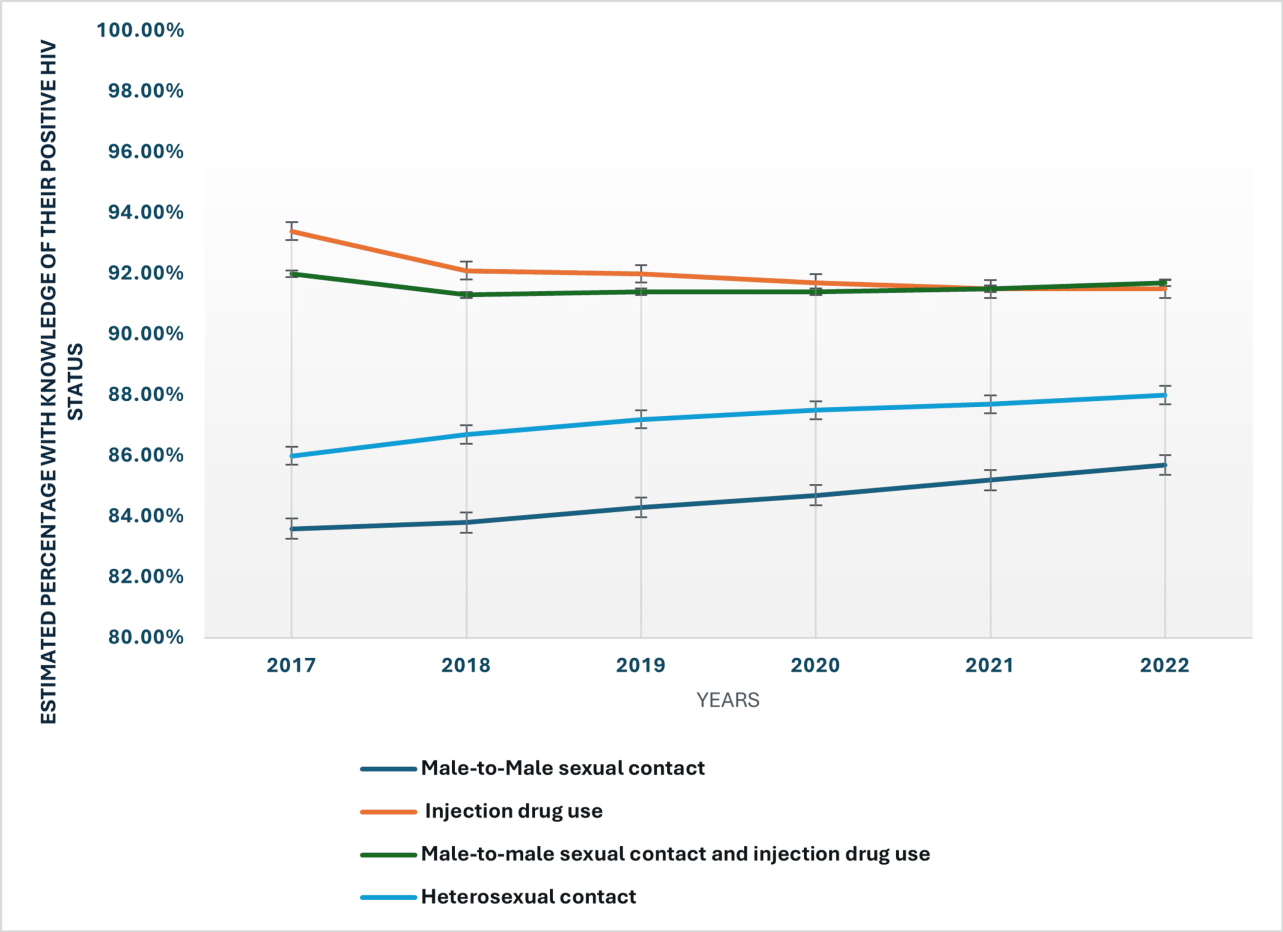
Modes of transmission and the knowledge percentage for each of positive HIV status

The mode of HIV transmission also influences the likelihood of individuals knowing their HIV status. By 2022, individuals who contracted HIV through heterosexual contact had an awareness rate of 88%, while those with male-to-male sexual contact had a lower rate of 85.70%. Those who contracted HIV through injection drug use had a high rate of knowledge at 91.50%, though this has slightly decreased since 2017. The consistent awareness among those with injection drug use transmission may reflect targeted efforts in harm reduction programs. The modes of transmission and the percentage of those with awareness of their positive HIV status for each mode have been presented in Figure 2.

HIV medical care within one month of diagnosis trend-based analysis

From 2017 to 2022, the percentage of people with HIV receiving medical care within one month of diagnosis showed an overall increase, rising from 20,630 (77.80%) to 20,630 (81.60%). The actual number of individuals linked to care fluctuated, peaking at 27,479 in 2019 before declining to 23,419 in 2020, likely due to the coronavirus disease 2019 (COVID-19) pandemic. By 2022, the number of people receiving care had increased to 29,753, indicating progress in linkage efforts. While there has been overall progress, the data shows a slight regression in the last two years, indicating potential challenges in maintaining consistent access to timely care. The national goal is to increase this linkage to care to 95% by 2025, an ambitious target given the current trends [3,9,10]. The data from 2017 to 2022 focusing on the percentage of newly diagnosed individuals who received HIV medical care within one month of diagnosis have been presented in Table 2.

**Table 2:** People with HIV who received HIV medical care within one month of diagnosis -: Not available

Label	Variable	2017	2018	2019	2020	2021	2022
National data	Number of people with HIV who received HIV medical care within one month of diagnosis	26,517	26,858	27,479	23,419	27,535	29,753
	Percentage of people with HIV who received HIV medical care within one month of diagnosis	20,630 (77.80%)	21540 (80.20%)	22340 (81.30%)	19297 (82.40%)	22551 (81.90%)	29,753 (81.60%)
Based on gender	National, Male (sex)	77.70%	80.30%	81.60%	82.40%	82%	81.90%
	National, Female (sex)	78.20%	79.80%	80.20%	82.30%	81.80%	80.20%
Based on age	National, 13-24	74.60%	77.20%	79%	80%	80.20%	80.30%
	National, 25-34	76.70%	79.50%	80.80%	82.30%	81.70%	81.40%
	National, 35-44	79.30%	82.30%	82.40%	82.50%	82.20%	82.10%
	National, 45-54	81%	81.60%	83.30%	84.50%	83.40%	82.20%
	National, 55-64	-	83.20%	82.80%	83.90%	84%	82.90%
	National, 65+	-	81.40%	85.50%	86.30%	83.80%	82.10%

When analyzing the linkage to care by gender, the data reveals a relatively consistent trend between males and females, with males slightly outperforming females in most years. In 2022, 81.90% of males received care within one month of diagnosis, compared to 80.20% of females. The slight fluctuations in these percentages over the years suggest that both genders face similar barriers and facilitators in accessing timely care, but the marginal differences point to a need for gender-sensitive approaches to further improve these rates.

Age plays a significant role in linkage to HIV care. The data shows that older age groups, particularly those aged 45-54, have consistently higher rates of linkage to care, with 82.20% of individuals in this age group receiving care within one month in 2022. Younger individuals, particularly those aged 13-24, have lower rates, with only 80.30% receiving timely care in 2022. Although there has been an improvement in the younger age group since 2017, the persistently lower rates suggest that younger individuals may face unique challenges in accessing care, such as stigma, lack of awareness, or healthcare access issues.

**Figure 3:**
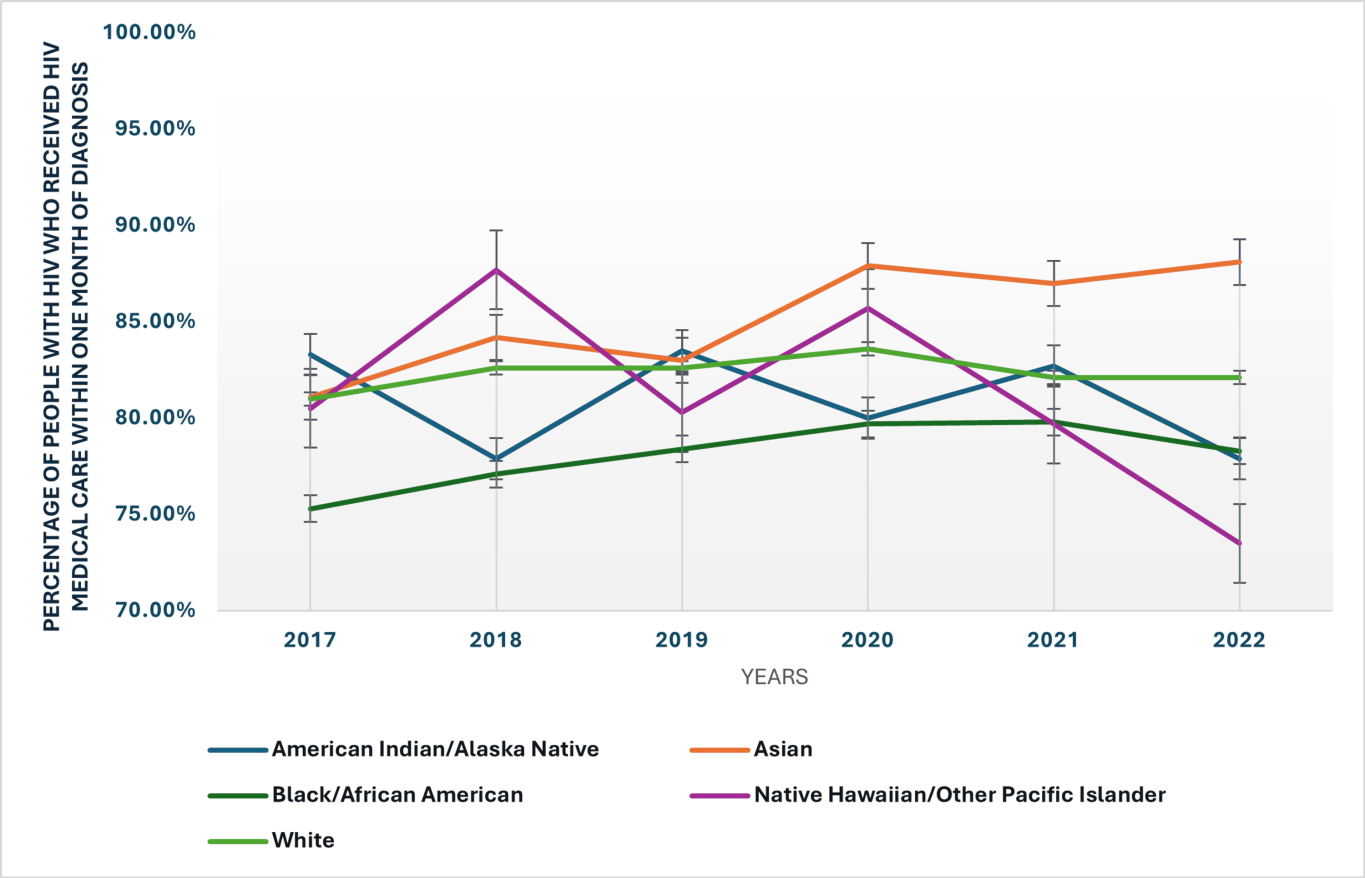
Racial and ethnic disparities in people with HIV who received HIV medical care within one month of diagnosis

The data also highlights significant racial and ethnic disparities in linkage to care. Asians consistently show the highest rates of timely linkage to care, reaching 88.10% in 2022. In contrast, American Indians/Alaska Native persons and Black/African American individuals have some of the lowest rates, with 77.90% and 78.30% linked to care within one month in 2022, respectively. These disparities indicate systemic challenges in healthcare access and suggest the need for targeted interventions to address the specific barriers faced by these communities. The racial and ethnic disparities in individuals with HIV who received HIV medical care within one month of diagnosis have been presented in Figure 3.

**Figure 4:**
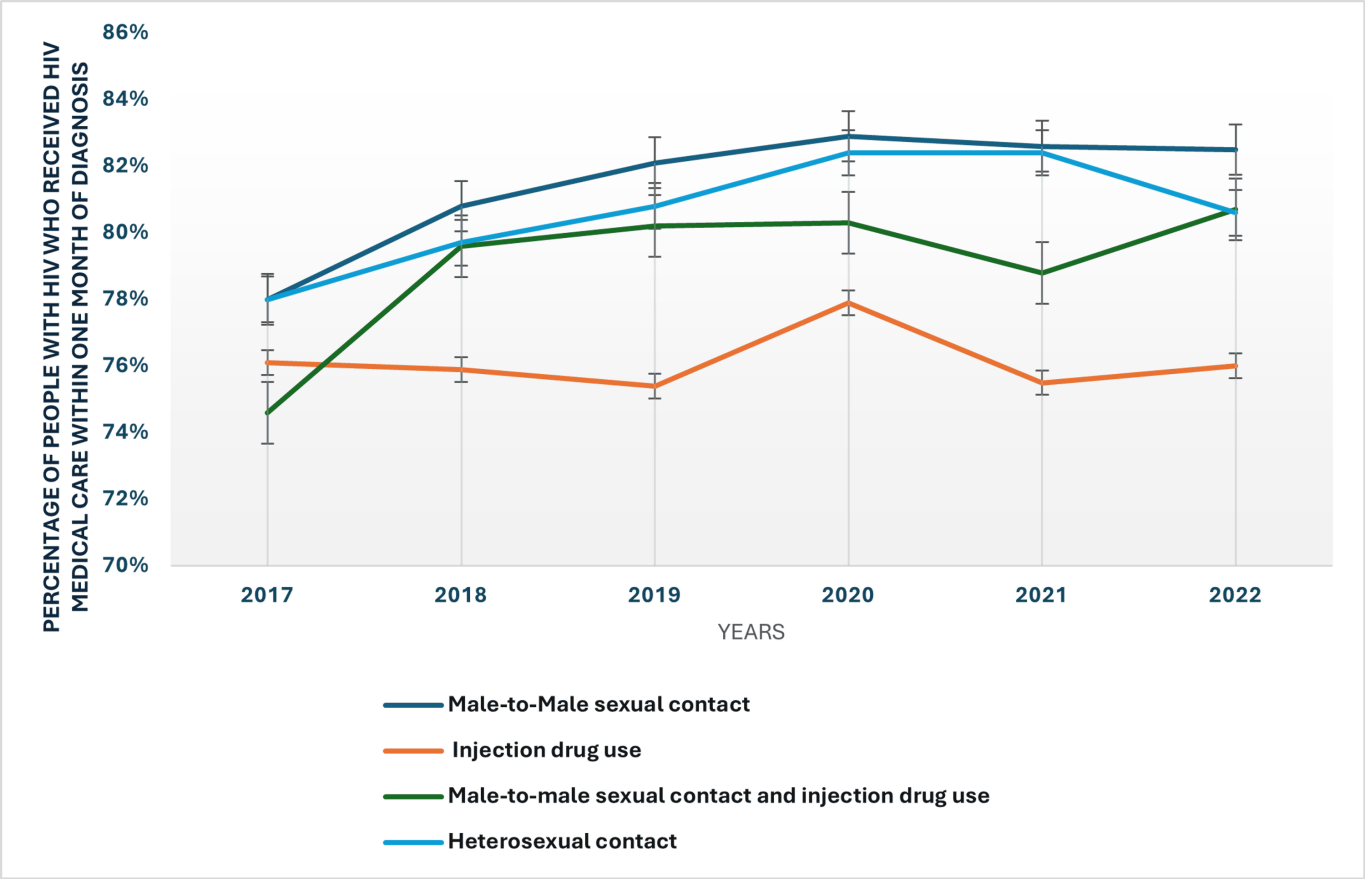
Modes of transmission in people with HIV who received HIV medical care within one month of diagnosis

The mode of HIV transmission is another factor influencing linkage to care. Individuals with male-to-male sexual contact as the mode of transmission show relatively high linkage rates, with 82.50% receiving care within one month in 2022. In contrast, those with injection drug use as the mode of transmission have consistently lower rates, with only 76% linked to care in 2022. This highlights the need for tailored strategies to improve access to care for individuals who acquire HIV through injection drug use, as they may face additional barriers such as stigma or lack of resources. The modes of transmission in people with HIV who received medical care within one month of diagnosis have been presented in Figure 4.

## Discussion

The analysis of knowledge status and HIV linkage to care utilizing the AHEAD National Database offers a detailed overview of the current HIV care landscape in the United States. By comparing our findings with prior research, we can discern progress, identify gaps, and pinpoint areas that require further attention.

From 2017 to 2022, our analysis indicates a steady increase in the percentage of people living with HIV who are aware of their positive status, rising from 85.8% to 87.2%. This incremental improvement resonates with findings from previous studies conducted globally. For example, data from the WHO in 2023 similarly noted an upward trend, revealing that 86% of individuals living with HIV were aware of their status [4,10-13]. Such findings suggest that sustained efforts to enhance testing and awareness campaigns produce favorable outcomes.

Further evidence supporting this trend can be found in the research conducted by Shanaube et al., who reported an increase in knowledge status within the African context. The HPTN 071 (PopART) for Youth (P-ART-Y) study, from 2013 to 2018 in 21 communities across Zambia and South Africa, assessed the efficacy of various HIV prevention strategies. The communities were divided into three intervention arms: arm A received a comprehensive prevention intervention including universal ART, arm B followed local prevention guidelines, and Arm C served as the standard care group. The results revealed significantly higher knowledge of HIV status in arms A (78.2%) and B (76.0%) compared to arm C (32.9%) [13]. These findings underscore the potential of structured interventions in enhancing awareness of HIV status.

However, these statistics reflect progress and illuminate persistent disparities among different demographic groups. Notably, individuals aged 13-24 displayed consistently lower rates of awareness regarding their HIV status, with only 56.3% of this age group reporting awareness in 2022. This statistic aligns with previous studies that highlighted lower HIV awareness rates among younger populations [14-17]. Such disparities indicate an urgent need for targeted outreach and education initiatives tailored to younger individuals. These initiatives should aim to dismantle barriers such as stigma, misinformation, and limited access to testing services, ensuring that younger populations receive the necessary support to understand their health status.

In terms of linkage to care, the percentage of individuals with HIV who received medical care within one month of diagnosis rose from 77.8% in 2017 to a peak of 82.4% in 2020, before slightly declining to 81.6% in 2022. This positive trajectory aligns with findings from recent national reports, which also noted a gradual increase in linkage to care rates [4]. Organizations such as the WHO and the Global Fund have set ambitious goals to end the HIV epidemic by 2030, aiming for 95% of people living with HIV to be diagnosed, treated, and achieve viral load suppression by 2025. As of 2023, these figures stood at 86%, 89%, and 93%, respectively [4]. For example, a study conducted by the CDC in 2021 indicated that approximately 80% of newly diagnosed individuals were linked to care within 30 days [16]. The slight decline observed in 2022 may reflect disruptions stemming from the COVID-19 pandemic, corroborating findings from other studies that documented interruptions in healthcare services during this time [18-20].

Meyer et al. performed a systematic review to evaluate the pandemic's impact on HIV service engagement, treatment adherence, and viral suppression. Their analysis, encompassing 26 studies across national, state, and city levels within all CDC HIV surveillance regions, revealed mixed effects. The pandemic resulted in reduced HIV healthcare visits, increased provider cancellations, and challenges in prescription refills. Nevertheless, some studies have seen telehealth emerging as a pivotal tool for maintaining access to care and enhancing retention. However, disparities in access to telehealth resources were evident, disproportionately affecting certain HIV-positive populations [20].

Moreover, the mode of HIV transmission has been shown to influence linkage to care. Individuals who acquire HIV through male-to-male sexual contact tend to have higher linkage rates than those whose transmission occurred via injection drug use. This disparity aligns with previous research indicating that individuals acquiring HIV through injection drug use face additional barriers to care, such as stigma and limited access to harm reduction services [4,21]. Tailored interventions for people who inject drugs, such as needle exchange programs and integrated substance use treatment, are critical for enhancing care linkage within this demographic.

The stigma surrounding HIV continues to significantly impede both prevention and treatment efforts, particularly among men who have sex with men. Babel et al. conducted a scoping literature review exploring the impact of stigma on HIV-related outcomes in the United States. Their findings revealed that stigma serves as a substantial barrier to engagement in both prevention and treatment, affecting both HIV-negative and HIV-positive individuals. Internalized stigma among HIV-positive MSM was linked to diminished treatment engagement, particularly pronounced among those residing in the Southern United States who exhibited higher tendencies toward risky sexual behaviors. Additionally, perceived discrimination within healthcare settings negatively influenced awareness, particularly among HIV-negative Black MSM [22].

Strength and limitation

This analysis leverages the comprehensive AHEAD National Database, which offers an increasingly robust dataset with national coverage and highly detailed demographic breakdowns. The use of the AHEAD database in this study is a major strength, given that it provides valuable insights into trends over several years, facilitating the assessment of progress toward HIV care goals and identifying disparities across major demographic groups, including age, gender, and race/ethnicity. The big dataset offers the needed statistical power and enables the granular analysis of the trends related to care linkage.

However, the study's reliance on existing secondary data is considered a major limitation, given that it restricts its aptitude to aptly account for the significant contextual factors that might affect the outcomes of care linkage. For example, socioeconomic status is an important determinant of healthcare quality and access but has not been fully captured within the analyzed datasets. Consequently, geographical barriers, including urban versus rural access to healthcare, might also play an important role in the linkage rates, even though they have not been sufficiently addressed in the analyzed data. Such unmeasured variables are prone to introduce biases while limiting the study findings' generalizability.

Furthermore, the data collected between 2020 and 2021 are highly prone to be influenced by the COVID-19 pandemic, which will cause major disruption of healthcare services globally. The COVID-19 pandemic might have adversely affected the access to HIV services, as well as the patient’s willingness and aptitude to seek care, resulting in the tilted linkage rates. Whereas this study offers vital insights, it is important to consider the likely impacts of such disruptions while interpreting the trends, given that they might fail to aptly reflect the characteristic care patterns external to the COVID-19 pandemic context. Therefore, prospective studies need to account for such limitations by integrating more detailed geographic and socioeconomic data and adjusting for the pandemic-linked disruptions to ascertain a comprehensive understanding of the dynamics of care linkage.

Implications for public health strategies

The study findings have highlighted several areas in which prospective studies and public health intervention may be aptly reinforced to enhance the outcomes of HIV care and realize the 95% national linkage goal to care by 2025. In this regard, it is recommended that targeted outreach programs, especially for underserved populations, including racial/ethnic minorities and younger persons, be developed and executed. Prospective studies should also explore innovative outreach methods, such as digital health interventions, to enhance care engagement among such groups. Public health interventions are prone to gain from partnerships with various community-based organizations that such populations trust, thereby ensuring the resonation and accessibility of the messages.

Still, the integration of HIV care with other social and healthcare services presents the potential to enhance patient care outcomes through tackling the different barriers to care services. Prospective studies should concentrate on the evaluation of the efficiency of the various integrated care models, including the models that combine HIV care and treatment with substance use, primary care, and mental health services. Moreover, prospective studies should also evaluate the scalability of such integrated models within divergent healthcare contexts and how technology, including telemedicine, might facilitate more coordinated and comprehensive care. In this regard, it is recommended that the existing disparities in HIV care should be addressed through public health interventions that focus on the populations that continue to experience considerable disparities in HIV care. To attain this, prospective studies should assess the root causes of such disparities and design interventions customized to marginalized populations' socioeconomic and cultural settings. This entails the study of the effects of discrimination, stigma, and socioeconomic aspects on HIV care engagement, as well as the testing interventions that aim to minimize such barriers. Improving access to culturally competent care and effectively addressing the various social determinants of health, including employment and housing, must be at the core of public health interventions.

Consequently, for future practice implications, it is noteworthy that implementing evidence-based interventions, including integrating digital tools to improve patient engagement and incorporating telemedicine in HIV treatment and care, will be important. Future studies must continue to assess the efficiency of such tools in populations that are adversely affected and identify the best practices regarding their (tools) incorporation into routine HIV care. Furthermore, developing novel partnerships between technology organizations and public health organizations is recommended as such partnerships are likely to provide newer solutions to the challenge of access and delivery of HIV care.

## Conclusions

This analysis of the AHEAD National Database underscores significant progress in both knowledge of HIV status and linkage to care over recent years. Despite the decline in new infections and increased awareness, disparities persist among younger individuals and racial minorities, emphasizing the need for targeted interventions. The rising percentage of people receiving care within one month of diagnosis reflects improvements in healthcare accessibility and outreach efforts. However, continued focus on education, stigma reduction, and tailored support is essential to achieve the goal of optimal HIV care for all populations. As we move toward future public health objectives, addressing these disparities will be crucial in improving health outcomes and enhancing overall care for individuals living with HIV.
